# A single treatment with a fluralaner injectable suspension (Bravecto^®^ injectable) provides 1-year efficacy against *Rhipicephalus sanguineus* sensu lato and *Ctenocephalides felis* in dogs

**DOI:** 10.1186/s13071-024-06535-8

**Published:** 2024-10-26

**Authors:** Marie-Kristin Raulf, Katharina Raue, Anna Schwarz, Ivo Petersen, Eva Zschiesche, Lea Heinau, Christina Strube

**Affiliations:** 1 Institute for Parasitology, Centre for Infection Medicine, University of Veterinary Medicine Hannover, Buenteweg 17, 30559 Hanover, Germany; 2 MSD Animal Health Innovation GmbH, Schwabenheim, Germany

**Keywords:** Isoxazoline, Injectable, Injection, Ectoparasites, Flea, Tick, Long-acting, Year-round efficacy, Year-round protection

## Abstract

**Background:**

*Rhipicephalus sanguineus* sensu lato (s.l.) and *Ctenocephalides felis* are among the most important year-round ectoparasites of dogs. The persistent efficacy of one treatment with fluralaner injectable suspension (Bravecto^®^ 150 mg/ml powder and solvent for suspension for dogs, referred to as Bravecto^®^ injectable) was investigated in a negative-controlled, randomised, partially blinded 12-month laboratory study.

**Methods:**

A total of 20 dogs were randomly allocated to two equal groups (treatment and control). Treatment-group dogs were injected subcutaneously on study day 0 with the investigational veterinary product at the recommended dose of 15 mg fluralaner/kg body weight (0.1 mL/kg), whereas the control group dogs received saline solution (0.1 mL/kg). Each dog was infested with 50 (25 female, 25 male) adult *R. sanguineus* s.l. and 100 adult *C. felis* 2 days before treatment, 5 and 28 days after treatment, and then once monthly for a 12-month period. Live tick and flea counts were performed 48 h after treatment or subsequent infestation, respectively. Efficacy was determined by comparing arithmetic means of the treatment group tick and flea counts with those of the control group. Infestation was considered adequate if at least 25.0% of ticks and 40.0% of fleas were recovered from at least six dogs in the control group at the respective assessment times.

**Results:**

Adequate *R. sanguineus* s.l. and *C. felis* infestations of control group dogs were observed at each time point. Arithmetic mean treatment group values were significantly lower than those of the control group at all time points. The immediate efficacy when treating existing infestations of *R. sanguineus* s.l. and *C. felis* (infestation 2 days before treatment), was 49.7% and 89.7%, respectively. The persistent efficacy against post-treatment re-infestations was 94.4–100% against *R. sanguineus* s.l. and 92.2–100% against *C. felis*. Seven dogs in the control group developed flea allergy dermatitis due to the repeated re-infestations over the study period, whereas no dogs in the treatment group were affected. No clinically relevant side effects were observed over the entire study period.

**Conclusions:**

The fluralaner injectable suspension (Bravecto^®^ injectable) provides 1 year of efficacy against *R. sanguineus* s.l. and *C. felis* infestations in dogs following a single treatment, allowing once-yearly treatment, which can significantly improve owner compliance with year-round protection of dogs.

**Graphical Abstract:**

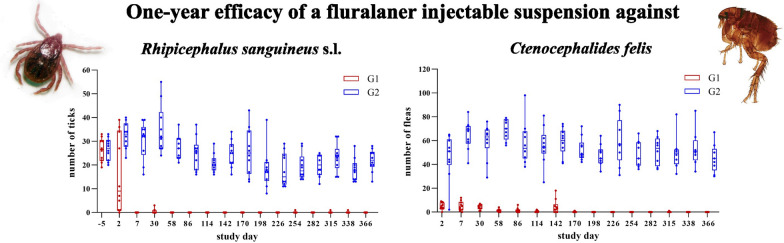

**Supplementary Information:**

The online version contains supplementary material available at 10.1186/s13071-024-06535-8.

## Background

Ticks and fleas are among the most relevant ectoparasites infesting dogs worldwide because of their clinical impact, relevance in the transmission of vector-borne pathogens and ubiquitous distribution. The brown dog tick *Rhipicephalus sanguineus* sensu lato (s.l.) is the most widespread tick of domestic dogs, with a worldwide distribution, being mainly found in warm climatic zones [1, 2]. However, import into colder climates such as Central Europe by dogs originating from or travelling to its main distribution area is common, with 40 import events reported in the UK from 2012 to 2016, and 26 events in Germany from 2019 to 2021 [3, 4]. As an endophilic tick, *R. sanguineus* s.l. mainly colonises indoor areas, being found on carpets, walls and furniture, but is also capable of an exophilic lifestyle in peri-domiciliary environments upon warm climate conditions and the availability of suitable hosts [5]. In polar, subpolar and certain temperate climatic zones, persistent outdoor populations cannot establish, but ticks rather exclusively inhabit houses, kennels or animal shelters as well as boardings, even leading to severe household infestations [6–10].

*Ctenocephalides felis* is commonly known as the cat flea, but it also frequently infests a variety of other hosts and represents one of the most important ectoparasites of domesticated dogs [11]. Within 48 h after host infestation and initial blood feeding, female *C. felis* produce eggs, which develop to adults in a few weeks under suitable environmental conditions. Unfed adult fleas can survive approximately 6 months in the environment in their pre-emergent state within the cocoon until a suitable host passes by [12]. The wide host range, endophilic life cycle and environmental persistence of *C. felis* enable its ubiquitous distribution all over the world. In Europe, veterinary practice-level surveys indicate high flea infestation rates of dogs, such as 14.1% in Hungary, up to 14.4% in the UK, 17.9% in Italy and up to 39.1% in Cyprus [11, 13–16], of which the majority is believed to be caused by *C. felis* rather than other flea species [11]. Discomfort to animals and humans is primarily caused by local bite reactions and itching, and importantly, episodic exposure can lead to flea allergy dermatitis (FAD) [17]. Furthermore, *C. felis* serves as an intermediate host for the tapeworm *Dipylidium caninum*, posing a risk not only to dogs and cats but also to humans owing to its zoonotic potential [18].

Both *R. sanguineus* s.l. and *C. felis* represent ectoparasites that pose year-round infestation risks to dogs because of their endophilic nature, thus rendering the need for reliable and effective control measures. In this context, adequate protection can only be achieved by good pet owner compliance, which may be substantially influenced by the frequency of treatment intervals, i.e. the duration of efficacy of veterinary products [19]. Recently, a fluralaner injectable suspension (Bravecto^®^ 150 mg/ml powder and solvent for suspension for injection for dogs; Merck Animal Health, Rahway, NJ, USA; in the following referred to as Bravecto^®^ injectable) has been registered and licensed in Australia [19, 20], New Zealand, the European Union and several Latin American countries, being the first ectoparasiticide with full-year efficacy against *R. sanguineus* s.l., *C. felis* and other ectoparasite species after a single treatment. In this formulation, a crystalline form of fluralaner allows the systemic absorption of the active ingredient from the injection site with slow elimination from plasma and a lack of extensive metabolism, wherefore effective levels can be maintained over a long-term period of 12 months [21]. Hence, the frequency of treatment can be reduced, simplifying owner compliance, reducing the risk of missed treatments and subsequently elevating canine health owing to seamless year-round protection against ticks and fleas [19]. The present study evaluated the long-term efficacy of fluralaner injectable suspension (Bravecto^®^ injectable) against European strains of *R. sanguineus* s.l. and *C. felis* in dogs as part of the application for marketing authorisation in the EU.

## Methods

### Study design

**Fig. 1:**
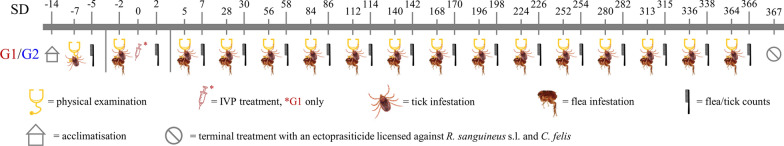
Overview of the design of the confirmation study to assess the immediate and persistent efficacy of an injectable fluralaner suspension (Bravecto^®^ injectable; IVP, recommended dose of 15 mg fluralaner/kg BW; G1). The control group (G2) received a placebo (0.9% saline solution). SD: study day

The persistent efficacy over a period of 12 months (52 weeks) of fluralaner injectable suspension (Bravecto^®^ 150 mg/ml powder and solvent for suspension for injection for dogs; Merck Animal Health, Rahway, NJ, USA; in the following referred to as Bravecto^®^ injectable) at the recommended dose of 15 mg/kg body weight (BW) against *R. sanguineus* s.l. and *C.* *felis* in dogs was evaluated in a negative-controlled, randomised and partially blinded efficacy study. The investigational veterinary product (IVP, Bravecto^®^ injectable) was administered subcutaneously as a single dose. Dogs were experimentally infested with both *R. sanguineus* s.l. and *C. felis* 2 days before treatment, 5 and 28 days after treatment, and then once a month over a 12-month period. A detailed study design is shown in Fig. 1. The study was performed in accordance with VICH guideline 9 “Good Clinical Practice” [22] and the European Medicines Agency’s (EMA) “Guideline for the Testing and Evaluation of the Efficacy of Antiparasitic Substances for the Treatment and Prevention of Tick and Flea Infestation in Dogs and Cats” [23].

### Study dogs

Twenty-four purpose-bred female (*n* = 12, intact) and male (*n* = 12, neutered) Beagle dogs were obtained from a commercial breeder. Of these, ten females and ten males at the age of approximately 12 months with BW ranging from 7.5 to 11.7 kg (mean 9.5 kg) were included in the study. For inclusion, dogs had to be clinically healthy, approximately 12 months of age at study day (SD) 0, susceptible to *R. sanguineus* s.l. infestation based on pre-treatment tick counts (SD–7, see below) and not treated with fluralaner within the past 180 days or with other drugs with long- or short-acting activity within the past 90 days that could interfere with the establishment of experimental tick or flea infestations. All dogs were acclimatised to the husbandry conditions at the Institute for Parasitology, University of Veterinary Medicine Hannover, Germany, for 7 days prior to first assessments.

The study dogs were kept pairwise in environmentally controlled indoor compartments with adjacent outdoor runs accessible during daytime, allowing a view of a green area or the university’s campus. The indoor area was equipped with raised resting areas and toys, and the outdoor runs with concrete pipes, raised resting areas with stairs and toys for jumping, playing, resting and observing. During infestation only, dogs were kept individually by dividing the indoor compartment, which prevented physical but allowed audio-visual and olfactory contact. On all other days, dogs were kept in groups of two dogs per group. The dogs received an age-appropriate commercial dog diet at recommended rates and water ad libitum and were trained with treats for handling procedures. After completion of the study, dogs were referred to private owners by animal mediation agencies.

### Group allocation and treatment regimes

Based on the pre-treatment *R. sanguineus* s.l. infestation on SD −7 and the respective assessment on SD −5, 24 dogs were ranked by descending order of live tick counts. The 4 dogs with the lowest tick counts were excluded from the study, and the remaining 20 dogs were grouped into ten blocks of 2 dogs each. Within each block, dogs were randomly allocated to experimental groups (G) 1 and 2 via a computer-generated randomisation list. Detailed information on the homogeneity of study groups according to age, weight and sex is listed in Supplementary Table 1.

The dogs in G1 were treated with a single subcutaneous injection of the IVP (Bravecto^®^ injectable) at the recommended dose of 15 mg fluralaner/kg BW (0.1 mL/kg) on SD 0. Dogs of G2 served as control and received a subcutaneous placebo injection (sterile 0.9% saline solution) using the same dose volume (0.1 mL/kg). Injection sites of all dogs were examined for abnormal signs, including erythema, heat and pain, scored as 0 = no reaction, 1 = slight reaction, 2 = moderate reaction or 3 = severe reaction. Swelling was categorised as visible or not, and if present, as palpable, hard or soft. Injection-site observations were conducted before treatment, at 10 min up to 1 h after treatment and on SD 1, 3, 4, 7, 10 and 14. For study release, dogs of both groups were treated according to regulatory requirements with an ectoparasiticide licensed against *R. sanguineus* s.l. and *C. felis* (Exspot^®^, Merck Animal Health, Rahway, NJ, USA).

### Health monitoring

General health observations were carried out once daily by qualified animal attendants. Upon abnormalities and if considered necessary, veterinarians performed comprehensive veterinary examination of affected dogs. Each dog was physically examined by a veterinarian for enrolment, before treatment and at each tick/flea infestation event to ensure good health of the study dogs.

### Infestations and assessments

Ectoparasites used for infestations were field isolates of *R. sanguineus* s.l. and *C. felis* collected in Europe. The *R. sanguineus* s.l. isolate was collected in 2017 from an apartment situated in Berlin, Germany. The isolate was maintained by a commercial breeder and purchased for this study. *Ctenocephalides felis* was isolated in 2020 from a cat in Hannover, Germany, and fleas were maintained by infestation of cats. Both strains complied with the "Guideline for the Testing and Evaluation of the Efficacy of Antiparasitic Substances for the Treatment and Prevention of Tick and Flea Infestation in Dogs and Cats" [23].

While dogs were infested with ticks only on SD −7 for study allocation, infestations with both ticks and fleas were carried out on SD −2 for assessment of immediate efficacy and on SDs 5, 28, 56, 84, 112, 140, 168, 196, 224, 252, 280, 313, 336 and 364 for assessment of persistent efficacy (Fig. 1). The dogs were sedated for infestations by intramuscular injection of 40 µg/kg BW medetomidine to provide at least 30–60 min for the ticks and fleas to disperse over the body and into the hair coat. At each infestation, approximately 50 (± 4) viable, unfed, adult *R. sanguineus* s.l. (approximately 25 females and 25 males) and 100 (± 4) viable, unfed adult *C. felis* were directly applied to the fur on the torso of each dog.

Ticks and fleas were counted by blinded study personnel at 48 ± 4 h after infestation on SD −5 (ticks only) and on SDs 7, 30, 58, 86, 114, 142, 170, 198, 226, 254, 282, 315, 338 and 366 (ticks and fleas), except for SD 2 where assessment was performed 96 h ± 4 h after infestation owing to treatment on SD 0 (Fig. 1). Dogs were examined for ticks and fleas by pushing the hair manually using the thumb, fingers or forceps to expose attachment sites on the skin, followed by gentle removal with special tick removal devices or forceps and, in the case of fleas, also with the fingers. Ticks that were recovered were collected, counted and categorised according to their general status, i.e. live or dead, and their attachment status, i.e. attached or free, and stored in a 50 ml tube to ensure that they were not counted twice. Recovered fleas were also categorised as live or dead, and drowned in soapy tap water to ensure that collected fleas were not counted twice. After completion of visual examination, dogs were combed with a fine-toothed flea comb (approximately 11–13 teeth/cm) for an additional period of at least 5 min to recover fleas and ticks that might have been missed visually. The combing procedure consisted of overlapping strokes from the front (whole head, ears, neck etc.) to the back including the tail, the lateral sides including the legs, the chest, and the ventral sides (armpits, belly, and inner side of legs). All areas were combed several times as fleas tend to flee from combed areas. Special attention was paid to the predilection sites such as hair whorls located beneath the ears and the caudal legs, the armpits, the belly, the croup area and the base of the tail.

### Statistical analysis and persistent efficacy

Statistical analysis was performed using the software package SAS^®^ (version 9.4; SAS Institute Inc., Cary, NC, USA).

According to the recommendations of the EMA and the World Association for the Advancement of Veterinary Parasitology (WAAVP), at least six dogs per group are required to sufficiently evaluate the efficacy of insecticides and acaricides in dogs and cats [23, 24]. Accounting for the likelihood of FAD development with shortfalls or complications in the control group due to the long-term duration of the study, ten dogs were included in each study group. Efficacy evaluation was considered adequate if at least 25.0% (13 ticks) of the tick infestation dose and at least 40.0% (40 fleas) of the flea infestation dose were recovered on at least six dogs of the control group at the respective assessment time points.

The primary efficacy criterion was the reduction of tick/flea counts in the treatment group (G1) compared with the control group (G2). Reduction of tick/flea counts were used to evaluate the percentage persistent efficacy of the treatment group according to the recommendations for controlled tests described in the EMA "Guideline on Statistical Principles for Veterinary Clinical Trials" [25] by Abbott’s formula:

$${\text{Efficacy reduction }}\left[ \% \right]\, = \,\frac{{{\text{M}}_{{\text{C}}} - {\text{M}}_{{\text{T}}} }}{{{\text{M}}_{{\text{C}}} }} \times 100$$

where M_C_ is the arithmetic mean (AM) of total live ticks/fleas in the control group (G2) and M_T_ is the AM of total live ticks/fleas in the treatment group (G1).

The validity of the efficacy results was confirmed by a statistical comparison of the live tick/flea counts in the treatment group (G1) with those of the control group, using a two-sided linear mixed model including the study group as a fixed effect and the randomisation block as a random effect.

Additionally, persistent percentage efficacy based on geometric means (GM) was calculated. To allow the calculation in case of zero counts, the GM was calculated as follows:

$$x_{g} = \left( {\prod\limits_{i = 1}^{n} {(x_{i} + 1)} } \right)^{\frac{1}{n}} - 1$$

In all analyses, a *P*-value of ≤ 0.05 was considered statistically significant.

## Results

### Inclusion criteria and safety assessment

All 24 dogs met the criteria for inclusion in the study. Based on the pre-treatment *R. sanguineus* s.l. counts assessed on SD −5, the four dogs with the lowest tick counts were excluded, so that 20 dogs were included in the study.

No abnormal signs at the injection sites were observed. A total of five serious adverse events, i.e. a benign cutaneous histiocytoma, urolithiasis and three observations of a severe FAD, were reported in the control group (G2). No serious adverse events were reported in the treatment group (G1). All adverse events were regarded as unrelated to treatment. Most frequently, vomiting after sedation occurred on 63 occasions in 14 dogs, of which 38 events were reported in nine dogs of the treatment group (G1) and 25 events in five dogs of the control group (G2). While seven dogs of the control group (G2) exhibited FAD, with three dogs developing a severe manifestation on later time points, none of the dogs of the treatment group (G1) was affected.

### Adequacy of infestation

Because of severe FAD even after concomitant medication with prednisolone, two dogs of the control group (G2) were not infested with fleas on certain time points, i.e. one dog on SD 280–336 and one dog on SD 224–364. Moreover, the same animal was not infested with both ticks and fleas on SD 56 owing to urolithiasis-related surgery. Thus, less data were available for efficacy evaluation. Nevertheless, the minimum requirement for adequacy of infestation was maintained at all assessment time points since at least 25.0% *R. sanguineus* s.l. and 40.0% *C. felis* were retained on at least six dogs of the untreated control group (G2) 48 h after infestation. Detailed tick and flea counts of the control group (G2) are listed in Table 1 (ticks) and Table 2 (fleas).

**Table 1:** Live tick counts in the dogs of the investigational veterinary product (IVP)-treated group (Bravecto^®^ injectable, recommended dose of 15 mg fluralaner/kg BW; G1) and of the control group (0.9% saline; G2) and adequacy of infestation of the dogs of the control group (0.9% saline; G2) infested with 50 (± 4) viable, unfed adult *Rhipicephalus sanguineus* sensu lato

SD	G1			G2			
	Min.	Max.	AM	Min.	Max.	AM	Adequately infested dogs
2	0	39	16.1	23	40	32.4	10/10
7	0	0	0.0	16	39	30.6	10/10
30	0	3	0.5	24	55	34.8	10/10
58	0	0	0.0	21	37	27.3	9/9
86	0	0	0.0	16	37	24.8	10/10
114	0	0	0.0	16	29	20.7	10/10
142	0	0	0.0	16	34	24.6	10/10
170	0	0	0.0	13	43	25.7	10/10
198	0	0	0.0	8	39	18.6	8/10
226	0	0	0.0	11	29	18.0	7/10
254	0	1	0.1	14	29	19.3	10/10
282	0	0	0.0	12	25	19.8	9/10
315	0	1	0.1	15	32	23.2	10/10
338	0	1	0.1	13	28	17.9	10/10
366	0	0	0.0	13	28	21.9	10/10

*SD* study day, *Min.* minimum, *Max.* maximum, *AM* arithmetic mean

**Table 2:** Live flea counts in the dogs of the investigational veterinary product (IVP)-treated group (Bravecto^®^ injectable, recommended dose of 15 mg fluralaner/kg BW; G1) and of the control group (0.9% saline; G2) and adequacy of infestation of the dogs of the control group (0.9% saline; G2) infested with 100 (± 4) viable, unfed adult *Ctenocephalides felis*

SD	G1			G2			
	Min.	Max.	AM	Min.	Max.	AM	Adequately infested dogs
2	0	9	4.8	2	65	46.5	8/10
7	0	12	5.1	41	84	65.3	10/10
30	1	7	4.2	29	76	59.6	9/10
58	0	4	1.3	56	79	69.4	9/9
86	0	6	1.4	38	98	58.2	9/10
114	0	2	0.6	25	81	55.7	9/10
142	0	18	4.5	41	74	59.2	10/10
170	0	1	0.2	42	72	52.4	10/10
198	0	0	0.0	34	64	47.1	9/10
226	0	0	0.0	31	90	59.1	7/9
254	0	0	0.0	36	66	48.4	7/9
282	0	0	0.0	36	68	51.5	6/8
315	0	1	0.1	32	82	50.0	6/8
338	0	0	0.0	34	85	53.3	7/8
366	0	0	0.0	30	67	45.6	6/9

*SD* study day, *Min.* minimum, *Max.* maximum, *AM* arithmetic mean

### Efficacy

**Fig. 2:**
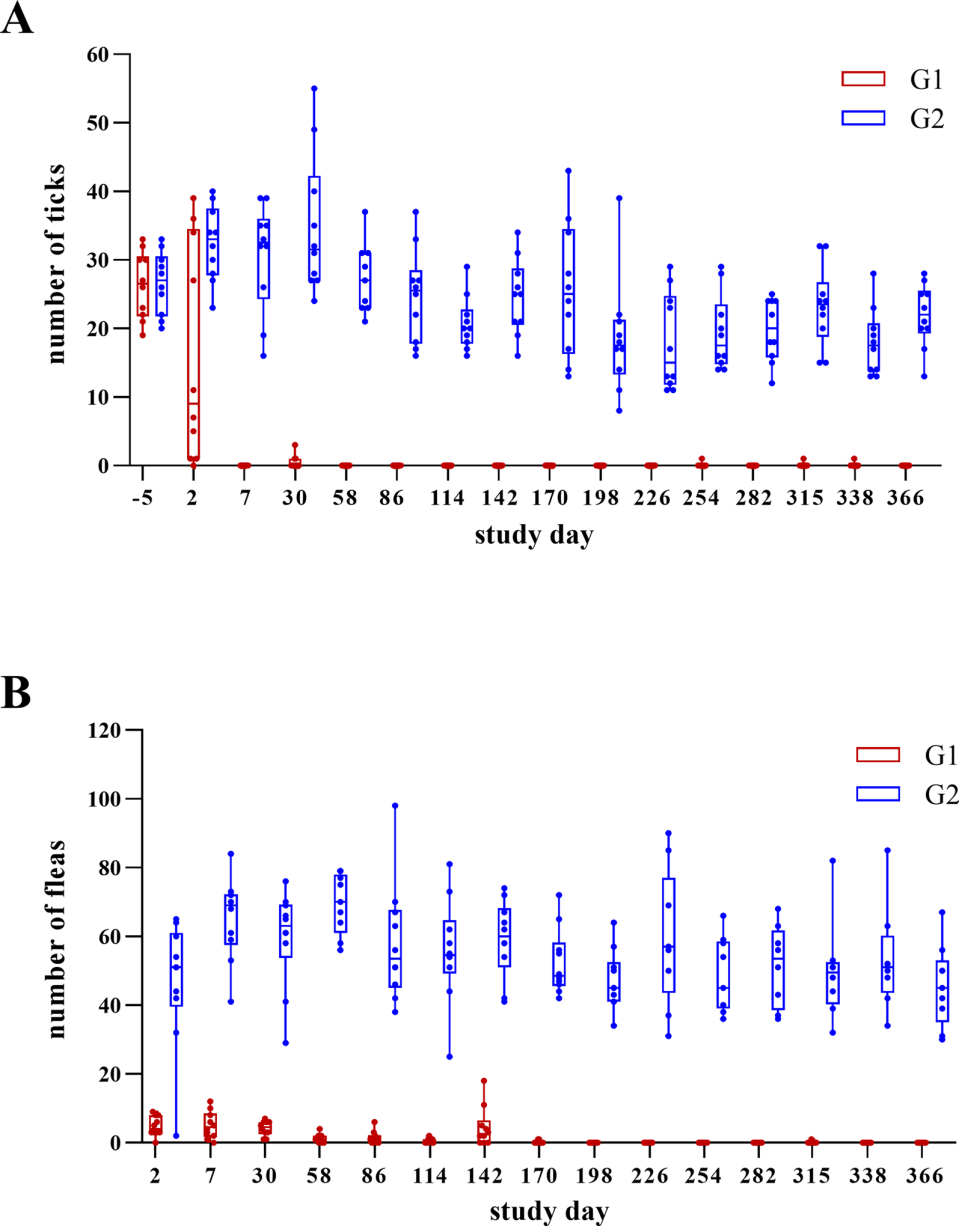
Live tick (**A**) and flea (**B**) counts of the dogs of the investigational veterinary product (IVP)-treated group (Bravecto^®^ injectable, recommended dose of 15 mg fluralaner/kg body weight; G1) and the control group (0.9% saline; G2). Ends of the boxes represent the 25th and 75th percentiles with a line at the median and error bars defining the 10th and 90th percentiles

In the untreated control group (G2), the AM of live *R. sanguineus* s.l. ranged from 17.9 to 34.8 with a slight decrease over the course of the study. From SD 2 to SD 170, AM constantly exceeded 20.0 ticks. Thereafter, AMs predominantly varied between 17.9 and 19.8, only slightly exceeding 20.0 ticks on two occasions. In contrast, live *R. sanguineus* s.l. AM of the treatment group (G1) mainly ranged from 0.0 to 1.0 (Fig. 2A). Accordingly, tick efficacy was > 94.0% over the whole year, except shortly after treatment on SD 2, where efficacy had only reached 49.7% owing to a live tick AM of 16.3 in the treatment group (G1) (Table 3). Efficacy based on GM is listed in Supplementary Table 2.

**Table 3:** Arithmetic mean (AM) of live tick counts in the dogs of the investigational veterinary product (IVP)-treated group (Bravecto^®^ injectable, recommended dose of 15 mg fluralaner/kg BW; G1) and of the control group (0.9% saline; G2) infested with 50 (± 4) viable, unfed adult *Rhipicephalus sanguineus* sensu lato

SD	Tick AM G1	Tick AM G2	Efficacy (%)	*F*-value	*P*-value
2	16.3	32.4	49.7	9.2	0.0076
7	0.2	30.6	99.4	148.0	< 0.0001
30	0.5	34.8	98.6	122.2	< 0.0001
58	0.3	27.3	98.9	347.1	< 0.0001
86	0.0	24.8	100	132.2	< 0.0001
114	0.4	20.7	98.1	277.5	< 0.0001
142	0.0	24.6	100	198.5	< 0.0001
170	0.3	25.7	98.8	66.4	< 0.0001
198	0.6	18.6	96.8	49.4	< 0.0001
226	1.0	18.0	94.4	55.1	< 0.0001
254	0.2	19.3	99.0	119.1	< 0.0001
282	0.1	19.8	99.5	183.4	< 0.0001
315	0.3	23.2	98.7	166.1	< 0.0001
338	0.1	17.9	99.4	136.4	< 0.0001
366	0.0	22.1	100	230.3	< 0.0001

*SD* study day

For *C. felis*, the AM of live fleas varied between 40.0 and 69.4 in the control group (G2), whereas those of the treatment group (G1) ranged from 0.0 to 5.1 (Fig. 2B). As for *R. sanguineus* s.l., live flea AMs in the control group (G2) slightly decreased over the course of the study. Until SD 226, AMs constantly exceeded 46.5, but ranged only from 40.0 to 43.6 fleas thereafter. On SD 2, 7, 30 and 142, efficacy was 89.7%, 92.2%, 93.0% and 92.4%, respectively. On all other time points, efficacy was > 97.0%, and from SD 170 onwards > 99.0% (Table 4). Efficacy based on GM is listed in Supplementary Table 3.

**Table 4:** Arithmetic mean (AM) of live flea counts in the dogs of the investigational veterinary product (IVP)-treated group (Bravecto^®^ injectable, recommended dose of 15 mg fluralaner/kg BW; G1) and of the control group (0.9% saline; G2) infested with 100 (± 4) viable, unfed adult *Ctenocephalides felis*

SD	Flea AM G1	Flea AM G2	Efficacy (%)	*F*-value	*P*-value
2	4.8	46.5	89.7	54.7	< 0.0001
7	5.1	65.3	92.2	222.3	< 0.0001
30	4.2	59.6	93.0	154.1	< 0.0001
58	1.3	69.4	98.1	591.4	< 0.0001
86	1.4	58.2	97.6	104.3	< 0.0001
114	0.6	55.7	98.9	130.3	< 0.0001
142	4.5	59.2	92.4	188.0	< 0.0001
170	0.2	52.4	99.6	290.6	< 0.0001
198	0.0	47.1	100	294.1	< 0.0001
226	0.0	59.1	100	90.7	< 0.0001
254	0.0	43.6	100	57.9	< 0.0001
282	0.0	41.3	100	29.8	< 0.0001
315	0.1	40.0	99.8	26.0	< 0.0001
338	0.0	42.6	100	26.4	< 0.0001
366	0.0	41.0	100	50.7	< 0.0001

*SD* study day

For both live ticks and fleas, AMs were significantly lower in the treatment group (G1) than in the control group (G2) at all post-treatment assessment time points (Tables 3 and 4).

## Discussion

A single subcutaneous injection of fluralaner suspension (Bravecto^®^ injectable) at the minimum recommended dose of 15 mg/kg BW effectively controlled European *R. sanguineus* s.l. and *C. felis* field isolates in experimentally infested dogs over a period of 1 year. Hence, Bravecto^®^ injectable is the first registered ectoparasiticide providing year-long efficacy against both ticks and fleas on dogs following a single administration. The injectable fluralaner suspension was approved to be persistently active against *R. sanguineus* s.l. from 4 days through 12 months after treatment [21]. Thus, dogs being at risk of acquiring *R. sanguineus* s.l. infestation should be thoroughly searched the first 3 days after treatment, and potentially occurring ticks should be removed. Furthermore, Bravecto^®^ injectable was registered and licensed in Europe, having an immediate and persistent *C. felis* killing activity for 12 months based on findings by Fisara and Guerino [19] showing an immediate efficacy against flea infestation. Moreover, from SD 7 until the end of the present study, the efficacy against fleas never dropped below 92.2% and on only two occasions, namely SD 30 and 142, was the efficacy below 95.0%. On all other 11 assessment time points, the efficacy against *C. felis* was above 95.0%, and from SD 170 until SD 366, efficacy ranged between 99.6% and 100%. The slight deviations from the efficacy threshold of 95.0% on SDs 2, 7, 30 and 142 were mainly based on a small number of outliers in the treated group with a maximum of 7–18 fleas on these occasions, while the maximum flea counts of the other days ranged from 0 to 6 specimen. These slight deviations are most likely attributed to inevitable biological variations during a study, e.g. differences in the feeding activities of the parasites and also taking into account that 2000 fleas were used for each infestation. Immediate and long-term efficacy above the threshold of 95% was shown in a study by Fisara and Guerino [19] with an Australian isolate of *C. felis*. Nevertheless, the results confirm the overall high long-term efficacy of the fluralaner injectable suspension against *C. felis* for a duration of 12 months as no fleas were found on any of the dogs of the treatment group even after 366 days.

A reduction in the number of adult fleas is a prerequisite for the effective management of FAD, which represents the most common clinical manifestation of flea infestations [26]. In this study, more than two-thirds of control dogs suffered from FAD, with pruritus, papules, erythema and alopecia being the predominant signs of FAD [17]. By contrast, none of the dogs of the treatment group was affected by FAD. It is conceivable that pruritus associated with elevated grooming behaviour had an effect on flea and tick counts [27], which slightly decreased during the study. Particularly in the dogs suffering from FAD, the number of retrieved ectoparasites even occasionally fell below the minimum requirement for adequacy of infestation. Nevertheless, at least six dogs of the control group were adequately infested at all assessment time points, wherefore requirements for the testing and evaluation of the efficacy of antiparasitic substances for the treatment and prevention of tick and flea infestation in dogs according to the WAAVP guideline were still met [24]. To account for reduced number of fleas due to excessive FAD-induced grooming behaviour, the flea retention rate for adequate efficacy evaluation in this study deviated from the 50.0% requested by the EMA guideline, as the guideline does not differentiate between short- and long-term studies and therefore likely does not account for FAD induction by a high number of repeated flea infestations.

The long-lasting efficacy of the injectable fluralaner suspension holds the potential to effectively control FAD, especially considering that episodic exposure particularly favours disease manifestation [28]. In this context, missed treatments with products that must be administered monthly or at 12-week intervals may lead to rapid re-infestation from contaminated environments or external sources, thus enabling episodic flea exposure. For instance, approximately 95% of the flea population, namely eggs, larvae and pupae, is located in the environment, whereas only 5% are adult fleas, which feed on vertebrate hosts and thus are noticed by pet owners [29]. Therefore, control measures against established flea infestations historically include extensive environmental decontamination, such as daily vacuuming, steam cleaning, regular washing or the use of suitable insecticides with a direct effect against immature stages [29]. Since these environmental stages tend to survive no longer than 6 months and newly emerging fleas from already contaminated environments or acquired from external sources will be killed on treated dogs before egg-laying, household flea populations can progressively be depleted by the administration of the injectable fluralaner suspension [19]. This also applies to indoor infestations with *R. sanguineus* s.l., being primarily found on carpets, walls and furniture and sometimes even leading to severe household infestations [5–10, 30]. With long-lasting protection against *R. sanguineus* s.l., established household infestations can at least be reduced as larvae and nymphs (which also preferentially feed upon dogs) can only survive for a short period of time in the environment, i.e. approximately 2−6 months [31–34]. Thus, the 12-month efficacy of the injectable fluralaner suspension holds the potential to break the life cycles of *C. felis* and *R. sanguineus* s.l. in contaminated environments.

A significant factor influencing effective ectoparasite control is pet owner compliance with treatment recommendations. Besides drug inefficacy and administration errors, non-adherence of owners to drug administration at regular intervals is considered to be a key factor contributing to ectoparasite control failure [35]. Many of the currently available products require monthly retreatment, thus often leading to poor compliance rates and reduced efficacy due to high risk of missed treatments [36]. For instance, a retrospective observational study of veterinary transactional records from animal hospitals in Spain including 30,738 dogs indicated that owners prescribed with a product that is effective against ectoparasites for 12 weeks protect their animals for longer periods than those prescribed with products that must be administered monthly. In this context, it is noticeable that most of these dog owners rather acquired only one instead of multiple ectoparasiticide doses, thus forgoing long-lasting protection [37]. Most dog owners seek general health check-ups of their animals at least once a year, mainly for vaccination as recommended by the WSAVA [38], offering an ideal opportunity for the yearly administration of the injectable fluralaner suspension by a veterinarian, thereby granting seamless, year-long ectoparasite protection of dogs. Thus, the likelihood of both missed treatments and administration errors can be drastically minimised, leading to substantially improved owner compliance with treatment recommendations for ectoparasite control [19].

## Conclusions

A single injection of fluralaner (Bravecto^®^ injectable) is effective against repeated experimental *R. sanguineus* s.l. and *C. felis* infestations of dogs for 1 year. The fluralaner injectable suspension was well tolerated and holds the potential to improve owner compliance, thus facilitating complete year-long protection of dogs against ticks and fleas to enhance canine health by reducing the risk of ectoparasite-mediated discomfort as well as transmission of vector-borne pathogens.

## Supplementary Information

Supplementary Material 1. Table 1: Homogeneity of the two study groups. Distribution of body weight (BW) and sex of the 20 study dogs of the investigational veterinary product (IVP)-treated group (Bravecto^®^ injectable, recommended dose of 15 mg fluralaner/kg BW; G1) and the control group (0.9% saline, G2). Std: standard deviation, Min: minimum, Max: maximum, SD: study day. Table 2: Geometric mean (GM) of live tick counts in the dogs of the investigational veterinary product (IVP)-treated group (Bravecto^®^ injectable, recommended dose of 15 mg fluralaner/kg BW; G1) and the control group (0.9% saline; G2) infested with 50 (± 4) viable, unfed adult *Rhipicephalus sanguineus* sensu lato. SD: study day. Table 3: Geometric mean (GM) of live flea counts in the dogs of the investigational veterinary product (IVP)-treated group (Bravecto^®^ injectable, recommended dose of 15 mg fluralaner/kg BW; G1) and the control group (0.9% saline; G2) infested with 100 (± 4) viable, unfed adult *Ctenocephalides felis*. SD: study day.

## Data Availability

Most data analysed during this study are included in the article. The remaining data from this clinical study are proprietary and maintained by MSD Animal Health.
