# Supplementary material for: A single treatment with a fluralaner injectable suspension (Bravecto® injectable) provides 1-year efficacy against Rhipicephalus sanguineus sensu lato and Ctenocephalides felis in dogs
Source: Parasit Vectors. 2024 Oct 26;17:438. doi: 10.1186/s13071-024-06535-8 (PMC11514862; doi:10.1186/s13071-024-06535-8)
Supplement: Supplementary file 1 — Supplementary Material 1. Table 1: Homogeneity of the two study groups. Distribution of body weight (BW) and sex of the 20 study dogs of the investigational veterinary product (IVP)-treated group (Bravecto® injectable, recommended dose of 15 mg fluralaner/kg BW; G1) and the control group (0.9% saline, G2). Std: standard deviation, Min: minimum, Max: maximum, SD: study day. Table 2: Geometric mean (GM) of live tick counts in the dogs of the investigational veterinary product (IVP)-treated group (Bravecto® injectable, recommended dose of 15 mg fluralaner/kg BW; G1) and the control group (0.9% saline; G2) infested with 50 (± 4) viable, unfed adult Rhipicephalus sanguineus sensu lato. SD: study day. Table 3: Geometric mean (GM) of live flea counts in the dogs of the investigational veterinary product (IVP)-treated group (Bravecto® injectable, recommended dose of 15 mg fluralaner/kg BW; G1) and the control group (0.9% saline; G2) infested with 100 (± 4) viable, unfed adult Ctenocephalides felis. SD: study day. [file 13071_2024_6535_MOESM1_ESM.docx]

**Supplementary Table 1:** Homogeneity of the two study groups. Distribution of body weight (BW) and sex of the 20 study dogs of the investigational veterinary product (IVP)-treated group (Bravecto^®^ injectable, recommended dose of 15 mg fluralaner/kg BW; G1) and the control group (0.9% saline, G2). Std: standard deviation, Min: minimum, Max: maximum, SD: study day.

|  | Study group | IVP treatment | No. of dogs | Mean | Std. | Min. | Median | Max. | female/male |
| --- | --- | --- | --- | --- | --- | --- | --- | --- | --- |
| BW [kg] | G1 | SD 0 | 10 | 9.7 | 1.2 | 8.3 | 9.5 | 11.7 | 5/5 |
|  | G2 | - | 10 | 9.3 | 0.8 | 7.5 | 9.5 | 10.1 | 5/5 |
|  | Total | | 20 | 9.5 | 1.0 | 7.5 | 9.5 | 11.7 | 10/10 |

**Supplementary Table 2:** Geometric mean (GM) of live tick counts in the dogs of the investigational veterinary product (IVP)-treated group (Bravecto^®^ injectable, recommended dose of 15 mg fluralaner/kg BW; G1) and the control group (0.9% saline; G2) infested with 50 (± 4) viable, unfed adult *Rhipicephalus sanguineus* sensu lato. SD: study day.

| **SD** | **Tick GM in G1** | **Tick GM in G2** | **Efficacy [%]** |
| --- | --- | --- | --- |
| 2 | 8.2 | 32.0 | 74.4 |
| 7 | 0.1 | 29.6 | 99.6 |
| 30 | 0.3 | 33.6 | 99.1 |
| 58 | 0.2 | 26.9 | 99.3 |
| 86 | 0.0 | 24.0 | 100 |
| 114 | 0.3 | 20.4 | 98.6 |
| 142 | 0.0 | 24.1 | 100 |
| 170 | 0.2 | 24.0 | 99.2 |
| 198 | 0.5 | 17.2 | 97.3 |
| 226 | 0.6 | 16.9 | 96.7 |
| 254 | 0.2 | 18.7 | 99.2 |
| 282 | 0.1 | 19.3 | 99.6 |
| 315 | 0.2 | 22.6 | 100 |
| 338 | 0.1 | 17.4 | 99.6 |
| 366 | 0.0 | 21.6 | 100 |

**Supplementary Table 3:** Geometric mean (GM) of live flea counts in the dogs of the investigational veterinary product (IVP)-treated group (Bravecto^®^ injectable, recommended dose of 15 mg fluralaner/kg BW; G1) and the control group (0.9% saline; G2) infested with 100 (± 4) viable, unfed adult *Ctenocephalides felis*. SD: study day.

| **SD** | **Flea GM in G1** | **Flea GM in G2** | **Efficacy [%]** |
| --- | --- | --- | --- |
| 2 | 3.9 | 37.7 | 89.5 |
| 7 | 3.8 | 64.2 | 94.1 |
| 30 | 3.7 | 57.7 | 93.6 |
| 58 | 1.0 | 69.0 | 98.5 |
| 86 | 0.9 | 56.2 | 98.4 |
| 114 | 0.5 | 53.5 | 99.1 |
| 142 | 2.5 | 58.2 | 95.8 |
| 170 | 0.2 | 51.7 | 99.7 |
| 198 | 0.0 | 46.4 | 100 |
| 226 | 0.0 | 56.2 | 100 |
| 254 | 0.0 | 31.8 | 100 |
| 282 | 0.0 | 24.0 | 100 |
| 315 | 0.1 | 21.6 | 99.7 |
| 338 | 0.0 | 22.8 | 100 |
| 366 | 0.0 | 29.9 | 100 |
